# Cytotoxic, DNA Cleavage and Pharmacokinetic Parameter Study of Substituted Novel Furan C-2 Quinoline Coupled 1, 2, 4-Triazole and Its Analogs

**DOI:** 10.2174/1874104501812010060

**Published:** 2018-05-31

**Authors:** Rajpurohit Anantacharya, Nayak D. Satyanarayan, Bhuvanesh Sukhlal Kalal, Vinitha Ramanath Pai

**Affiliations:** 1 Department of Pharmaceutical Chemistry, Kuvempu University, Post Graduate Centre, Kadur, 577548, Chikkamagalur Dist, Karnataka, India; 2 Department of Biochemistry, Yenepoya Medical College, Yenepoya University, Mangaluru, 575018, Karnataka, India.; 3 Yenepoya Research Centre, Yenepoya University, Mangaluru, 575018, Karnataka, India.

**Keywords:** Cancer, Melanoma cell line, Breast cancer cell line, ADMET, Cytotoxic, Quinoline

## Abstract

**Background:**

Furan, quinoline and triazoles are known for their wide spectrum biologically active molecules. A series of novel furan C-2 quinoline and 1, 2, 4-triazole (FQT) coupled hybrids were designed and synthesized to evaluate for their DNA cleavage and cytotoxic studies.

**Objectives:**

In this work we describe the synthesis and biological evaluation of furan C-2 quinoline coupled triazoles exposed for cytotoxic and DNA cleavage study.

**Methods:**

The electrophoretic DNA cleavage studies on λ-DNA (Eco-RI/Hinda-III double digest) using agarose gelelectrophoresis and the cytotoxic activity were carried out by MTT assay method.

**Results:**

The results revealed that, the molecules 7(a-o) did cleave the DNA completely with no trace of fragments at 100 µg concentration, on the other hand, cytotoxic assay was achieved by two different human cancer cell lines (melanoma cell line-A375 and breast cancer cell line MDA-MB 231). Among the synthesized compounds 7a, 7b, 7c and 7k exhibited potent cytotoxic activity with IC_50_ values ranging from 2.9, 4.0, 7.8 and 5.1 µg/ml against A375 and 6.2, 9.5, 11.3 and 7.3 µg/ml against, MDA-MB 231, respectively.

**Conclusion:**

In synthesized compounds 7(a-o) exhibited complete DNA cleavage at 100 µg/ml and the compounds 7a, 7b, 7c and 7k showed very less cytotoxic in nature. The structure activity relationship revealed that, the presence of halogen group/atoms at para position of phenyl ring remarkably enhanced the DNA cleavage and cytotoxic activities among the synthesized compounds.

## 1 INTRODUCTION

Cancer is the primary cause of human death worldwide [1]. Among all types of cancer, lung, breast, colorectal, stomach, and prostate cancer are the underlying causes for the majority of cancer death. Numerous anticancer agents have been designed, several of which exert antitumor effects by inducing apoptosis [2, 3]. Currently, in clinical use anticancer agents suffer from a number of drawbacks correlated to drugs’ associated side effects and/or tumors’ multi-drug resistance [4, 5]. Hence, it is still obviously of interest to search for new bioactive molecules having anticancer properties.

The chemistry of 1,2,4-triazoles and their coupled heterocyclic derivatives have received considerable attention owing to their synthetic and effective biological importance. For example, a large number of 1,2,4-triazoles have been incorporated into a wide variety of therapeutically interesting drug candidates possessing anticancer activities [6, 7], antimicrobial [8-12], anti-inflammatory [13] and analgesic [14]. Literature survey reveals that important chemotherapeutics, such as Vorozole, Letrozole and Anastrozole that consist of substituted 1,2,4-triazole ring, are currently being used for the treatment of breast cancer [15]. In addition to these important biological applications, coupled 1,2,4-triazoles are also of great utility in preparative organic chemistry as useful intermediates for the preparation some of the drug molecules. The amine group is ready to form hydrogen bonding and interact with target site [16], Where quinoline is a proven analogue for anticancer treatment [17].

In our search for new classes of potential anticancer agents we designed furan C-2 coupled quinoline derivative with 1,2,4-triazole nucleus and in continiuation of our research work on quinoline coupled oxygen and nitrogen heterocycles [18-20], we would like to report herein a facile and inexpensive procedure for the preparation of novel hybrid molecules 6-chloro-2-(furan-2-yl)-4-(5-phenyl-4*H*-1,2,4-triazol-3-yl) quinoline derivatives under mild conditions, and screened for *in vitro* anticancer, DNA cleavage and pharmacokinetic properties. We felt that there is a real need for the synthesis of new prototypes of compounds by the combin furan, quinoline and triazole moiety, which might end up as a potent analogs in the anticancer treatment.

## 2 EXPERIMENTAL

### 2.1 Materials and Methods

Chemicals used in the synthesis of compounds were purchased from Alfa Aesar, India and Spectrochem Pvt. Ltd. India. The solvents were of analytical grade. Melting points (M. Pt.) of the synthesized compounds were determined with the help of digital Raga digital melting point apparatus, Bengaluru, India and are uncorrected; Infrared data were recorded on a Bruker spectrophotometer using KBr pellets. ^1^H and ^13^C NMR spectra were recorded on Bruker AVANCE II 400 and 100 MHz instruments using DMSO-d6/CDCl_3_ as a solvent and TMS as an internal standard; chemical shifts are expressed as δ values (ppm). The J values are expressed in Hertz (Hz). Mass spectra (MS) were recorded in JEOL GCMATE II LC-Mass spectrometer using electron impact ionization (EI) technique. Analytical thin-layer chromatography (TLC) was performed on precoated TLC sheets of silica gel 60 F254 (Merck, Darmstadt, Germany), visualized by long and short wavelength UV lamps (356 and 254 nm). Chromatographic purifications were performed on silica gel (100-200 mesh, Merck, Germany).

#### 2.1.1 General Procedure for the Synthesis of Chloro 2-(1-Furan-2-yl) Quinoline-4-Carboxylic acid (3)

The compound (3) was synthesized by literature method [21] with slight modification. After completion, the reaction mixture was kept in an ice bath until solid mass of sodium salt of cinchonic acid is obtained. The obtained solid mass was filtered, further dissolved in water and acidified with acetic acid to get compound (3). The synthesized compound was purified by recrystalization using ethyl acetate as solvent. M. pt. 285-287^o^C.

#### 2.1.2 Procedure for the Synthesis of Chloro Methyl 2-(1-Furan-2-yl) Quinolone-4-Carboxylates (4)

Analog of 2-(1-furan-2-yl) quinoline-4-carboxylic acid was dissolved in sufficient quantities of methanol with catalytic amount of Conc. H_2_SO_4_ and kept for reflux on water for about 10-12 hrs. The reaction progress is checked by TLC. After completion, the reaction mixture was cooled to room temperature and poured onto the crushed ice to obtain solid mass which was filtered, washed with water, dried and recrystallized from petroleum ether (60-80).

#### 2.1.3 General Procedure for the Synthesis of Chloro 2-(Furan-2-yl) Quinoline-4 Carbohydrazide (5)

The mixture of methyl 2-(1-furan-2-yl) quinoline-4-carboxylate (0.5 mmol) and hydrazine hydrate (1 mmol) was taken into 30 ml of dry ethanol in a 50 ml round bottom flask and refluxed for 8-10 hrs. After completion of the reaction the resulting white solid mass was filtered off in hot condition and washed with cold ethanol followed by water to remove unreacted hydrazine hydrate to obtain compound in pure form.

#### 2.1.4 General Procedure for the Synthesis of Substituted 2-(Furan-2-yl)-4-(5-Phenyl-4*H*-1,2,4-Triazol-3-yl) Quinoline 7(a-o)

The solution of 2-(furan-2-yl) quinoline-4 carbohydrazide (0.0038 mol) in acetic acid and add a equimolar amount of ammonium acetate was added followed by the addition of substituted aldehyde (0.0038 mol) and the mixture was stirred for 2 to 3 hrs at room temperature. The solution was then neutralized with liq. ammonia and the product obtained was filtered, washed with water and recrystalized from ethanol to yield the pure products.

### 2.2 Spectral Details

#### 2.2.1 6-Chloro-2-(Furan-2-yl)-4-(5-Phenyl-4*H*-1,2,4-Triazol-3-yl) Quinoline (7a)

Yield: 79%. M. Pt. 244-246^o^C; IR (KBr), cm^-1^ 1493 (C=C), 3065 (C-H), 3405 (N-H); ^1^H NMR (DMSO-d_6,_ 400 MHz, δ ppm): 12.31 (s, 1H), 8.39 (s, 1H), 8.20-8.21 (d, 1H, *J=4* Hz), 8.10-8.12 (t, 1H, *J=8* Hz), 8.012-8.015 (d, 1H, *J=1.2* Hz), 7.851-7.857 (d, 1H, *J=2.4* Hz), 7.80-7.81 (t, 2H), 7.490-7.495 (d, 4H), 7.23-7.25 (d, 1H, *J=8* Hz); ^13^C NMR (DMSO-d_6,_ 100 MHz, δ ppm): 162.1, 152.2, 149.3, 148.4, 146.4, 145.8, 140.0, 133.9, 131.7, 131.1, 130.5, 128.9, 127.3, 126.6, 124.0, 123.9, 117.0, 116.6, 112.9, 112.2; Calculated mass: 372.80g/mol; MS (m/z): 375.20 g/mol (M+H).

#### 2.2.2 6-chloro-4-[5-(4-Chlorophenyl)-4*H*-1,2,4-Triazol-3-yl]-2-(Furan-2-yl) Quinoline (7b)

Yield: 84%. M. Pt. 244-246^o^C; IR (KBr), cm^-1^ IR (KBr), cm^-1^ 3429 (N-H), 1659 (C=N), 1596 (C=C), 3059 (C-H), 824(C-Cl); ^1^H NMR (DMSO-d_6,_ 400 MHz, δ ppm): 12.39 (s, 1H), 8.38 (s, 1H), 8.200-8.206 (d, 1H, *J=2.4* Hz), 8.09-8.12 (d, 1H, *J=12* Hz), 8.010-8.013 (d, 1H, *J=1.2* Hz), 7.872-7.878 (d, 1H, *J=2.4* Hz), 7.81-7.82 (t, 2H, *J=4* Hz), 7.55-7.57 (d, 1H, *J=8* Hz), 7.47-7.48 (d, 1H, *J=4* Hz), 7.23-7.26 (d, 1H, *J=12* Hz), 6.743-6.748 (d, 1H, *J=2* Hz): ^13^C NMR (DMSO-d_6,_ 100 MHz, δ ppm): 162.1, 152.2, 148.3, 148.0, 145.8, 135.0, 132.8, 131.8, 131.3, 131.1, 129.0, 128.2, 123.9, 117.1, 112.9, 112.2; Calculated mass: 407.25 g/mol; MS (m/z): 409.14 g/mol (M-H).

#### 2.2.3 6-Chloro-4-[5-(4-Fluorophenyl)-4*H*-1,2,4-Triazol-3-yl]-2-(Furan-2-yl) Quinoline (7c)

Yield: 81%. M. Pt. 248-250^o^C; IR (KBr), cm^-1^ 3437 (N-H), 1657 (C=N), 1508 (C=C), 3069 (C-H), 744 (C-Cl), 1235 (C-F); ^1^H NMR (DMSO, δ ppm): 12.29 (s,1H), 8.40 (s, 1H), 8.283-8.289 (d, 1H, *J=2.3* Hz), 8.125-8.129 (d, 1H, *J=1.6* Hz), 8.06-8.08 (d, 1H, *J=16*Hz), 7.807-7.809 (d, 2H, *J= 0.4* Hz), 7.70-7.71 (t, 1H, *J=4* Hz), 7.42-7.43 (d, 1H, *J=4* Hz), 7.20-7.22 (t, 2H, *J=8* Hz); ^13^C NMR (DMSO-d_6,_ 100 MHz) (δ ppm): 162.3, 152.2, 148.2, 148.1, 146.4, 144.6, 139.5, 132.1, 130.7, 130.7, 130.0, 129.3, 129.2, 124.0, 123.9, 116.4, 115.5, 115.3, 115.22, 112.44, 111.19; Calculated mass: 390.79 g/mol; MS (m/z): 392.0 g/mol (M+H).

#### 2.2.4 6-Chloro-4-[5-(2, 6-Difluorophenyl)-4*H*-1,2,4-Triazol-3-yl]-2-(Furan-2-yl) Quinoline (7d)

Yield: 78%. M. Pt. 256-258^o^C; IR (KBr), cm^-1^ 3417 (N-H), 1657 (C=N), 1547 (C=C), 3062 (C-H), 748 (C-Cl), 1238 (C-F); ^1^H NMR (DMSO-d_6,_ 400 MHz, δ ppm): 12.42 (s, 1H), 8.54 (s, 1H), 8.21-8.22 (d, 1H, *J=4* Hz), 8.10-8.12 (d, 1H, *J=8* Hz), 8.08-8.10 (d, 1H, *J=8* Hz), 8.01-8.03 (d, 1H, *J=8* Hz), 7.82-7.85 (t, 1H, *J=12* Hz), 7.51-7.52 (d, 1H, *J=4* Hz), 7.22-7.24 (t, 1H, *J=8* Hz), 6.98-7.01 (t, 1H, *J=12* Hz), 6.742-6.745 (d, 1H, *J=1.2* Hz): ^13^C NMR (DMSO-d_6,_ 100 MHz, δ ppm): 162.0, 161.7, 152.2, 148.4, 146.4, 145.8, 145.6, 139.8, 139.6, 131.8, 131.3, 131.1, 130.8, 124.0, 123.8, 117.1, 112.9, 112.5, 112.3, 111.8; Calculated mass: 409.0 g/mol; MS (m/z): 411.08 g/mol (M+2).

#### 2.2.5 6-Chloro-2-(Furan-2-yl)-4-[5-(4-Methoxyphenyl)-4*H*-1,2,4-Triazol-3-yl] Quinoline (7e)

Yield: 83%. M. Pt. 232-234^o^C; IR (KBr), cm^-1^ 3427 (N-H), 1606 (C=N), 1512 (C=C), 3069 (C-H), 748 (C-Cl), 1171 (C-O); ^1^H NMR (DMSO-d_6,_ 400 MHz, δ ppm): 12.18 (s, 1H), 8.32 (s, 1H), 8.201-8.206 (d, 2H, *J=2* Hz), 8.07-8.09 (t, 1H, *J=8* Hz), 8.00-8.04 (d, 1H, *J=16* Hz), 7.84-7.85 (d, 1H, *J=4* Hz), 7.72-7.74 (d, 2H, *J=8* Hz), 7.50-7.51 (d, 1H, *J=4* Hz), 7.04-7.07 (d, 1H, *J=12* Hz), 6.74-6.75 (t, 1H, *J=4* Hz), 3.83 (s, 3H); ^13^C NMR (DMSO-d_6,_ 100 MHz, δ ppm): 161.8, 152.2, 149.1, 148.4, 145.8, 131.7, 131.1, 129.0, 124.0, 116.9, 114.4, 112.9, 55.3 (o-methoxy carbon); Calculated mass: 402.83 g/mol; MS (m/z): 404.0 g/mol (M+H).

#### 2.2.6 4-{5-[6-Chloro-2-(Furan-2-yl) Quinolin-4-yl]-4*H*-1,2,4-triazol-3-yl}-*N*, *N*-Dimethylaniline (7f)

Yield: 80%. M. Pt. 268-270^o^C; IR (KBr), cm^-1^ 3423 (N-H), 1651 (C=N), 1595 (C=C), 3060 (C-H), 1231 (C-N), 746 (C-Cl); ^1^H NMR (DMSO-d_6,_ 400 MHz, δ ppm): 11.99 (s, 1H), 8.23 (s, 1H), 8.17 (s, 1H), 8.01-8.02 (d, 3H), 7.58-7.60 (d, 2H, *J=8* Hz), 7.50-7.51 (d, 1H, *J=4* Hz), 6.77-6.78 (d, 3H, *J=4* Hz), 2.99 (s, 6H); ^13^C NMR (DMSO-d_6,_ 100 MHz, δ ppm): 167.5, 161.9, 152.8, 152.2, 151.8, 150.4, 148.8, 146.8, 146.0, 143.0, 140.8, 132.0, 131.7, 131.2, 129.2, 128.3, 124.5, 121.5, 121.5, 117.3, 113.2, 112.5, 40.4; Calculated mass: 416.0 g/mol; MS (m/z): 418.14 g/mol (M+2).

#### 2.2.7 4-{5-[6-Chloro-2-(Furan-2-yl) Quinolin-4-yl]-4*H*-1,2,4-Triazol-3-yl}-*N*, *N*-Diethylaniline (7g)

Yield: 76%. M. Pt. 250-252^o^C; IR (KBr), cm^-1^ 2972.31 (N-H), 1521.84 (C=C), 821.68 (C-Cl); ^1^H NMR (DMSO-d_6,_ 400 MHz, δ ppm): 11.96 (s, 1H), 8.18-8.20 (d, 2H, *J=8* Hz), 8.08-8.10 (d, 1H, *J=8* Hz), 8.003-8.005 (d, 1H, *J=0.8* Hz), 7.83-7.84 (d, 1H, *J=4* Hz), 7.54-7.56 (d, 2H, *J=8* Hz), 7.50-7.51 (d, 1H, *J=4* Hz), 6.72-6.74 (d, 3H, *J=8* Hz), 3.34-3.37 (d, 4H, *J=12* Hz), 1.108-1.125 (t, 6H, *J=6.8* Hz); ^13^C NMR (DMSO-d_6,_ 100 MHz, δ ppm): 161.4, 152.2, 150.1, 149.2, 148.4, 146.4, 145.7, 140.4, 131.6, 131.2, 131.0, 129.1, 124.1, 120.1, 116.9, 112.8, 112.1, 111.0, 43.7, 12.4 (N N diethyl carbon); Calculated mass: 443.92 g/mol; MS (m/z): 444.0 g/mol (M+H).

#### 2.2.8 6-Chloro-2-(Furan-2-yl)-4-[5-(4-Methylphenyl)-4*H*-1,2,4-Triazol-3-yl] Quinoline (7h)

Yield: 74%. M. Pt. 242-244^o^C; IR (KBr), cm^-1^ 3196.05 (N-H), 1654.92 (C=N), 1544.98 (C=C), 744.52 (C-Cl); ^1^H NMR (DMSO-d_6,_ 400 MHz, δ ppm): 12.24 (s, 1H), 8.35 (s, 1H), 8.202-8.208 (t, 1H, *J=2.4* Hz), 8.09-8.11 (d, 1H, *J=8* Hz), 8.00-8.01 (t, 1H, *J=4* Hz), 7.81-7.84 (t, 1H, *J=12* Hz), 7.67-7.69 (d, 1H, *J=8* Hz), 7.47-7.48 (d, 1H, *J=4* Hz), 7.30-7.32 (d, 2H, *J=8* Hz), 7.05-7.07 (d, 1H, *J=8* Hz), 6.743-6.744 (d, 1H, *J=4* Hz), 2.50 (s, 3H): Calculated mass: 386.83 g/mol; MS (m/z): 389.0 g/mol (M+H).

#### 2.2.9 6-Chloro-2-(Furan-2-yl)-4-[5-(3, 4, 5-Trimethoxylphenyl)-4*H*-1,2,4-Triazol-3-yl] Quinoline (7i)

Yield: 77%. M. Pt. 276-278^o^C; IR (KBr), cm^-1^ 2918.30 (N-H), 1622.13 (C=N), 1577.77 (C=C), 1124.50 (C-O), 763.81 (C-Cl); ^1^H NMR (DMSO-d_6,_ 400 MHz, δ ppm): 12.19 (s, 1H), 8.31 (s, 1H), 8.16-8.19 (d, 2H, *J=12* Hz), 8.05-8.07 (t, 1H, *J=8* Hz), 7.44-7.46 (d, 1H, *J=8* Hz), 7.07 (s, 2H), 6.71-6.74 (d, 1H, *J=12* Hz), 6.52 (s, 1H), 3.86 (s, 6H), 3.72 (s, 3H); ^13^C NMR (DMSO-d_6,_ 100 MHz, δ ppm): 168.0, 162.4, 153.7, 152.6, 149.6, 148.8, 146.8, 146.2, 144.5, 140.0, 132.2, 131.7, 131.1, 129.8, 124.7, 124.3, 118.0, 117.4, 113.2, 112.6, 105.0, 104.2, 60.6, 55.7; Calculated mass: 463.00 g/mol; MS (m/z): 465.13 g/mol (M+2).

#### 2.2.10 4-{5-[6-Chloro-2-(Furan-2-yl) Quinolin-4-yl]-4*H*-1,2,4-Triazol-3-yl}-2-Methoxyphenol (7j)

Yield: 81%. M. Pt. 222-224^o^C; IR (KBr), cm^-1^ 3198.33 (N-H), 1653.00 (C=N), 1595.13 (C=C), 779.59 (C-Cl); ^1^H NMR (DMSO-d_6,_ 400 MHz, δ ppm): 12.3 (s, 1H), 8.26 (s, 1H), 8.18-8.20 (d, 2H, *J=8* Hz), 8.09-8.11 (d, 2H, *J=8* Hz), 7.842-7.848 (t, 1H, *J=2.4* Hz), 7.50-7.51 (d, 1H, *J=4* Hz), 7.37 (s, 1H), 7.12-7.14 (d, 1H, *J=8* Hz), 6.85-6.87 (d, 1H, *J=8* Hz), 6.773-6.778 (t, 1H, *J=2.0* Hz), 3.8 (s, 3H); ^13^C NMR (DMSO-d_6,_ 100 MHz, δ ppm): 152.2, 148.4, 131.7, 131.3, 131.0, 124.1, 116.9, 115.5, 112.8, 112.1, 55.5 (m-methoxy carbon); Calculated mass: 418.83 g/mol; MS (m/z): 421.00 g/mol (M+2H).

#### 2.2.11 6-Chloro-2-(Furan-2-yl)-4-[5-(1*H*-Indol-3-yl)-4*H*-1,2,4-Triazol-3-yl] Quinoline (7k)

Yield: 78%. M. Pt. 258-260^o^C; IR (KBr), cm^-1^ 3188.33 (N-H), 1651.07 (C=N), 1593.20 (C=C), 742.59 (C-Cl); ^1^H NMR (DMSO-d_6,_ 400 MHz, δ ppm): 12.09 (s, 1H, triazole N-H proton), 11.97 (s, 1H, indole N-H proton), 8.93 (s, 1H), 8.55 (s, 1H), 8.36-8.37 (d, 3H, *J=4* Hz), 8.22-8.25 (d, 1H, *J=12* Hz), 8.14-8.16 (d, 1H, *J=8* Hz), 8.010-8.015 (d, 2H, *J=2.0* Hz), 7.84-7.85 (d, 1H, *J=4* Hz), 7.80-7.82 (d, 1H, *J=8* Hz), 7.71-7.72 (d, 1H, *J=4* Hz); ^13^C NMR (DMSO-d_6,_ 100 MHz, δ ppm): 167.1, 152.3, 146.3, 145.7, 140.6, 137.1, 131.6, 131.2, 131.0, 130.8, 124.3, 124.1, 122.8, 122.0, 120.8, 120.6, 116.9, 116.6, 112.9, 112.1, 111.9, 111.8, 111.4; Calculated mass: 411.84 g/mol; MS (m/z): 415.20 g/mol (M+2H).

#### 2.2.12 6-Chloro-4-[5-(2-Chloroquinolin-3-yl)-4*H*-1,2,4-Triazol-3-yl]-2-(Furan-2-yl) Quinoline (7l)

Yield: 72%. M. Pt. 238-240^o^C; IR (KBr), cm^-1^ 3198.33 (N-H), 1641.42 (C=N), 1591.27 (C=C), 736.81 (C-Cl); ^1^H NMR (DMSO-d_6,_ 400 MHz, δ ppm): 12.46 (s, 1H), 8.30 (s, 2H), 8.24-8.25 (d, 1H, *J=4* Hz), 8.10-8.11 (d, 2H, *J=4* Hz), 7.71-7.73 (t, 1H, *J=8* Hz), 7.53-7.55 (d, 2H, *J=8* Hz), 7.34-7.35 (t, 1H, *J=4* Hz), 7.23-7.24 (t, 1H, *J=4* Hz), 6.78-6.79 (d, 2H, *J=4* Hz); ^13^C NMR (DMSO-d_6,_ 100 MHz, δ ppm): 162.2, 152.1, 148.5, 148.3, 147.3, 146.4, 145.8, 144.4, 139.5, 136.1, 132.0, 131.8, 131.3, 131.1, 129.1, 127.9, 127.6, 126.8, 125.7, 124.0, 117.1, 112.9, 112.2; Calculated mass: 458.29 g/mol; MS (m/z): 460.21 g/mol (M+2).

#### 2.2.13 6-Chloro-2-(Furan-2-yl)-4-[5-(2-Nitrophenyl)-4*H*-1,2,4-Triazol-3-yl] Quinoline (7m)

Yield: 80%. M. Pt. 248-250^o^C; IR (KBr), cm^-1^ 3188.34 (N-H), 1654.92 (C=N), 1544.98 (C=C), 717.52 (C-Cl); ^1^H NMR (DMSO-d_6,_ 400 MHz, δ ppm): 12.63 (s, 1H), 8.49 (s, 1H), 8.22-8.25 (d, 1H, *J=12* Hz), 8.10-8.11 (d, 2H, *J=4* Hz), 8.014-8.017 (d, 1H, *J=1.2* Hz), 7.85-7.86 (t, 2H, *J=4* Hz), 7.71-7.73 (t, 1H, *J=8* Hz), 7.50-7.52 (d, 2H, *J=8* Hz), 6.780-6.784 (t, 1H, *J=1.6* Hz): ^13^C NMR (DMSO-d_6,_ 100 MHz, δ ppm): 168.5, 162.7, 152.8, 148.8, 146.8, 146.0, 143.0, 140.8, 132.0, 131.7, 131.2, 129.2, 128.3, 124.5, 121.5, 117.3, 113.2, 112.5, 112.1, 40.4; Calculated mass: 417.80 g/mol; MS (m/z): 419.0 g/mol (M+H).

#### 2.2.14 6-Chloro-2-(Furan-2-yl)-4-{5-[2-(Trifluoromethyl) Phenyl]-4*H*-1,2,4-Triazol-3-yl} Quinoline (7n)

Yield: 79%. M. Pt. 228-230^o^C; IR (KBr), cm^-1^ 3190.26 (N-H), 1651.07 (C=N), 1546.91 (C=C), 754.17 (C-Cl); ^1^H NMR (DMSO-d_6,_ 400 MHz, δ ppm): 12.60 (s, 1H), 8.76 (s, 1H), 8.31-8.33 (d, 1H, *J=8* Hz), 8.23-8.24 (d, 1H, *J=4* Hz), 8.10-8.12 (d, 1H, *J=8* Hz), 8.02 (s, 1H), 7.81-7.83 (d, 3H, *J=8* Hz), 7.69-7.71 (d, 1H, *J=8* Hz), 7.530-7.538 (d, 1H, *J=3.2* Hz), 6.781-6.785 (t, 1H, *J=20* Hz); ^13^C NMR (DMSO-d_6,_ 100 MHz, δ ppm): 162.3, 152.1, 148.3, 146.4, 145.8, 144.2, 140.7, 139.5, 133.0, 131.8, 131.7, 131.3, 131.1, 130.5, 127.0, 126.0, 124.0, 123.8, 117.1, 112.9, 112.2; Calculated mass: 441.0 g/mol; MS (m/z): 443.09 g/mol (M+2).

### 2.2.15 15 3-{5-[6-Chloro-2-(Furan-2-yl) Quinolin-4-yl]-4*H*-1,2,4-Triazol-3-yl} Phenol (7o)

Yield: 81%. M. Pt. 264-266^o^C; IR (KBr), cm^-1^ 3192.19 (N-H), 1654.92 (C=N), 1543.05 (C=C), 1311.59 (C-F), 748.38 (C-Cl); ^1^H NMR (DMSO-d_6,_ 400 MHz, δ ppm): 12.25 (s, 1H), 9.69 (s, 1H), 8.29 (s, 1H), 8.20-8.21 (d, 1H, *J=4* Hz), 8.09-8.12 (d, 1H, *J=12* Hz), 7.966-7.969 (d, 1H, *J=1.2* Hz), 7.850-7.855 (t, 1H, *J=20* Hz), 7.51-7.52 (d, 1H, *J=4* Hz), 7.24-7.25 (d, 2H), 7.14-7.16 (d, 1H, *J=8* Hz), 6.884-6.889 (d, 1H, *J=2* Hz), 6.77-6.78 (d, 1H, *J=4* Hz); ^13^C NMR (DMSO-d_6,_ 100 MHz, δ ppm): 168.2, 162.4, 158.2, 157.9, 152.8, 149.8, 146.8, 146.0, 142.6, 140.4, 135.6, 132.2, 131.7, 130.4, 124.5, 119.5, 118.8, 117.9, 116.9, 113.5, 112.5; Calculated mass: 388.80g/mol; MS (m/z): 390.0 g/mol (M+H).

### 2.3 ADME-toxicity Prediction

The molecular descriptors of synthesized compounds 7(a-o) are predicted by pharmacokinetic parameters like absorption, distribution, metabolism, excretion and toxicity. The ADMET/SAR [22] helps to evaluate biologically active molecules and eliminate biologically poor molecules, active lead molecules which contain undesirable functional groups based on Lipinski rule. The statistical calculation for lead molecules includes surface area, geometry and fingerprint properties which help to understand biologically important end points. Aqueous Solubility (PlogS), Blood Brain Barrier Penetration (QlogBB), intestinal absorption (logHIA) [23] and hepatotoxicity, Caco-2 cell permeability (QPPCaco) also help to predict the toxicity of lead molecules with intraperitoneal, oral, intravenous and subcutaneous toxic effects. The *in-silico* study enables to decide the safety and efficacy of active molecules take up the molecule for in depth studies.

### 2.4 DNA Cleavage Study

The degree of DNA cleavage by the compounds was monitored by agarose gel electrophoresis method [24]. Agarose 0.25 gm was weighed and dissolved in 25 ml of tris acetate (TAE) buffer (50 mM, pH 8.0) and gel cassette was placed in the electrophoresis chamber inundated with TAE buffer. To this was loaded 20 µl of DNA sample along with bromophenol blue dye in 1:1 ratio with standard DNA marker. CT DNA was treated with analogs (40 µM, 2µl) followed by the dilution the buffer to a total volume of 20 µl. The samples after incubation at 37^o^C were loaded onto the wells.

Electrophoretic mobility was achieved by supplying 50 V of electricity for about 45 min. in TAE buffer. The gel along with platform was stained with 100 ml ethidium bromide (10 µg/ml) in sterile distilled water. Ethidium bromide binds to double stranded DNA by intercalation, because of steric hindrance for intercalation in covalently closed circular DNAs; it binds less than linear and open circles. After about 10-15 min. the platform and the gel were rinsed with distilled water and gel was gently placed onto the UV transilluminator. The DNA bands appeared on the gel determined the cleavage by the entitled molecules tested.

### 2.5 Cell Lines and Cell Culture

The human melanoma cell line, A375 and human breast cancer cell line MDA-MB231 were purchased from the National Centre For Cell Science (NCCS Pune, India), cultured in Dulbecco's modified Eagle's medium (DMEM) supplemented with 10% heat-inactivated fetal bovine serum, 2 mmol/l glutamine, 1% antibiotics solution (100 U/ml penicillin G and 100 mg/ml streptomycin). Cells were grown in 25 cm^2^ tissue culture flasks and maintained at 37^o^C in a humidified incubator with 5% CO_2_. All the consumables and plastic-wares were procured from Himedia, India.

#### 2.5.1 MTT Cell Viability Assay [18]

When the density reaches approximately 70% confluency the cells culture was trypsinized and 100 µl cell suspension (a density of 5000 cells/well) was added into 96-well plates and allowed to adhere overnight. Next day, the drugs were dissolved in DMSO and the final stock concentration of 1mg/ml was prepared in DMEM (final DMSO concentration was < 1%). A serial dilution of drugs, ranging from 320 μg/ml to 1.25 μg/ml, was prepared in DMEM, and cells were treated in triplicates. Untreated cells represented a control group. After 72 hrs of incubation, media was aspirated, 100 µl MTT (1 mg/ml) was added to each well and plate was incubated at 37^o^C for 4 hrs. After incubation, the media/MTT solution was aspirated and 100 μl DMSO was added to all the wells. Using multi-mode micro plate reader, a uniform shaking (300 rpm for 5 min) was applied to dissolve Formosan crystals and the absorbance was recorded at 570 nm. Cell viability was calculated using the following equation: (test-sample OD-blank control OD)/(untreated control OD-blank control OD) × 100%. A non-linear regression graph was plotted between cell survival (%) and log_10_drug concentration and the 50% minimum inhibitory concentration (IC50) was determined using Graph Pad Prism software.

## 3 RESULTS AND DISCUSSION

### 3.1 Chemistry

**Scheme (1):**
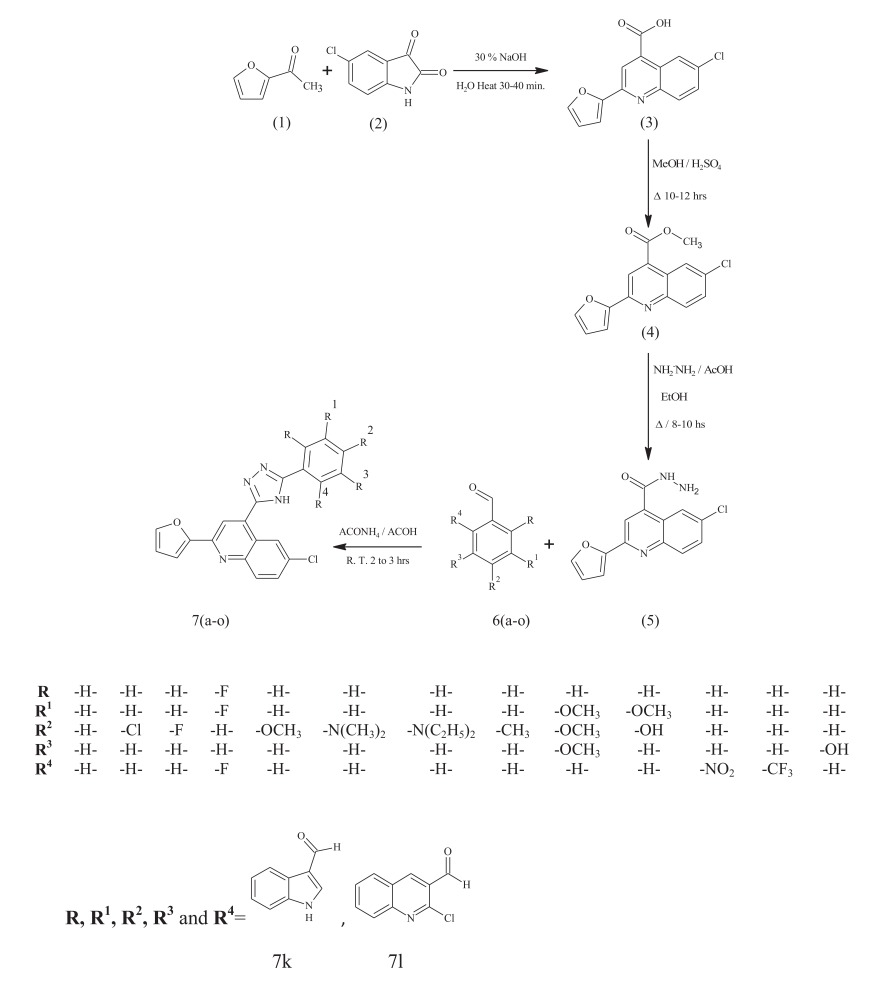
Synthesis of substituted 2-(1-furan-2-yl)-4-(5-phenyl-4*H*-1,2,4-triazol-3-yl) quinoline and its analogue 7 (a-o).

Substituted furan quinoline carboxylic acids **(3)** were prepared by coupling substituted isatins and 2-acetylfuran (Scheme **1**). Furan quinoline carboxylic acids were converted to their corresponding carboxylates **(4)** using methanol and catalytic amount of sulphuric acid. The carboxylates upon treatment with hydrazine hydrate resulted in carbohydrazide **(5)**, which upon intermolecular cyclization with substituted benzaldehydes and heterocyclic aldehydes **6(a-o)** in the presence of glacial acetic acid afforded novel 2-(1-furan-2-yl)-4-(5-phenyl-4*H*-1,2,4-triazol-3-yl) quinoline and its analogs **7(a-o)** (Scheme **1**). IR, ^1^H NMR, ^13^C NMR and mass spectral data are in well agreement with the proposed structures of the synthesized compounds. Formation of products was confirmed by spectroscopic analysis as in case compound 2-(1-furan-2-yl)-4-(5-phenyl-4*H*-1,2,4-triazol-3-yl) quinoline **7(a-o)**, the IR spectra indicated the absence of band around 3200 cm^-1^ due to the absence of -NH_2_ and observed band at 3405 cm^-1^ due to the presence of -NH group in triazole ring system.

Formation of product was further established by ^1^H NMR spectrum, here we can easily characterize the molecule by peak observed at 12.31 ppm (-NH). Furan, quinoline and phenyl protons resonated between the ranges of 8.21-7.23 ppm. Disappearance of NH_2_ proton of **7a** in proton NMR proved the ring closer by the formation of 1,2,4 triazole ring from **6a**.

This was further substantiated by the ^13^C NMR data of **7a**, exhibited signals at 162.1 and 152.2 ppm due to the C_2_ and C_5_ carbons of triazole. The aromatic carbons resonated signals in between 149.3-112.2 ppm which are in agreement with the expected values. Mass spectrum of **7a** displayed molecular ion peak at m/z 375.20 (M+2) g/mol, confirming its molecular weight. Rest of the compounds gave satisfactory spectroscopic data, which has been in accordance with their assigned structures.

### 3.2 *In Silico* ADMET (Absorption, Distribution, Metabolism, Excretion and **Toxicity) Profile**

The pharmacokinetic studies indicated that the compounds with poor bioavailability show less effectiveness against disease. To overcome this problem, predicting bioavailability properties will be of great advantage for drug development. Hence, using computer based methods like ADMET and SAR tools the molecular descriptors and drug likeliness properties were studied. The toxicity of the substituted 2-(furan-2-yl)-4-(5-phenyl-4H-1,2,4-triazol-3-yl) quinoline was predicted based on lethal dosage and functional ranges in different tissues. The mouse LD_50_ and probability of health effects were predicted using ACD / I-Lab 2.0 (guest). LD_50_ of potential compounds detect the cumulative potential of acute toxicity administered through oral, subcutaneous, intraperitoneal, intravenous and subcutaneous routes on mouse models. The analysis with test compounds on oral, subcutaneous, intraperitoneal and intravenous is lower when compared to reference molecule. The toxicity results suggest that the compounds 7a-o have less toxic effect in internal tissue and with no side effect (Table **1**).

The interpretation of tested compounds 7a-o was in acceptable range and hence, they can be used to make an oral formulation for absorption. The intestinal absorption (log_HIA_) and Caco-2 cell permeability (PCaco-2) are within the range of -2 poor absorption and +2 more absorption show that the compounds are more permeable in intestine and help in for transporting good drug metabolic compounds.

The reference range of -5 (poor) to +1 (good) and substrate inhibitor from 0 to 1 in which the reference and test compounds 7a-o show good activity with human intestinal absorption and metabolism. The aqueous solubility of compounds lies in the range of 0 (poor) to 2 (good) showing that all the molecules had good solubility. While the reference compound as well as test compounds came within acceptable range (Table **2**).

### 3.3 DNA Cleavage Study

**Fig. (1):**
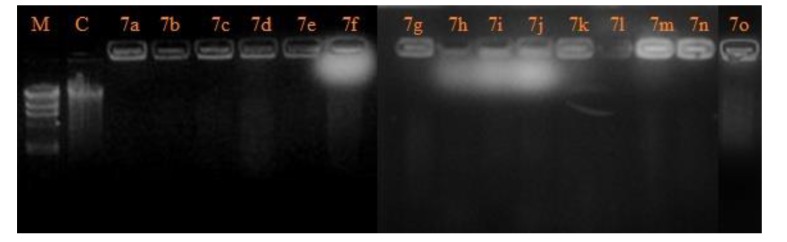
Photograph showing DNA cleavage on agarose gel electrophoresis by the derivatives of the synthesized compounds.

DNA has been the drug target for drug as it regulates many biochemical reactions occuring in the cellular system. Literature studies revealed that the clinical efficacies of many drugs correlate with their ability to induce enzyme mediated DNA cleavage. The loci present in the DNA are involved in various regulatory processes, such as gene expression, gene transcription, mutagenesis, and carcinogenesis [25] *etc.* In particular, designing of the compound having the ability to cleave DNA is utmost important not only from the primary biological point of view but also in terms of photodynamic therapeutic approach to develop potent drugs [26]. The compounds which were found to be active in CT-DNA cleavage were screened for their antiproliferative study. The DNA cleavage of furan quinoline triazole hybrids were studied by agarose gel electrophoresis method. The cleavage potential of the tested compounds were examined by comparing the bands appeared in control and test compounds at 100 µg concentration. The photograph (Fig. **1**) clearly demonstrates that compounds 7(a-o) did cleave the DNA completely, as no traces of DNA fragments were found. However, the ability of reactive intermediates involved in the DNA cleavage by the compounds has not been cleared. Control experiment does not reveal any significant cleavage of DNA even after prolong exposure of substrate.

### 3.4 *In Vitro* Cytotoxic Activity

The oxygen and nitrogen coupled heterocyclic compounds, prepared in this study, were evaluated according to standard protocols for their *in vitro* anticancer activity against two human cancer cell lines including cells derived from melanoma cell line, A375 and breast cancer cell line MDA-MB-231 [23]. All the IC_50_ values in micro gram/mililiter (µg/ml) are listed in Table **3** and Fig. (**2**). For comparison cisplatin was used as a standard cytotoxic drug, that showed the IC_50_ values of 1.3 and 5.1 µg/ml against melanoma cell line and breast cancer cell line, respectively. Four of the tested compounds: 7a (A375) 7b-c (MDA-MB-231) and 7k exhibited good cytotoxic effects with IC_50_ values of 2.9, 4.0, 7.8 and 5.1 µg/ml against A375; and 6.2, 9.5, 11.3 and 7.3 µg/ml against, MDA-MB 231, respectively. While rest of the compounds exhibited moderate to less cytotoxic against the cancer cell lines studied. The results revealed that, 7a, 7b, 7c and 7k can be taken up for future investigation as these molecules can be taken up as possible hits.

### 3.5 Structural Activity Relationship Study

There are few key features regarding structural requirements for these FQT hybrids 7(a-o) to exert their anticancer activity. Our initial strategy was to identify the key sub unit required for activity such as, furan (antibiotic properties of drugs), quinoline (nitrogen heterocycles -Anti cancer agents) and 1,2,4 triazole (active pharmacophore, which allows its derivatives to readily interact with diversity of enzymes and receptors in organisms). Further essential substituents like, [-N(C_2_H_5_)_2_, -N(CH_3_)_2_, -CH_3_ and -NH_2_ (electron donating), -OCH_3_ (electron releasing), -F, -Cl (halogen) and -NO_2_, -OH,(electron withdrawing)] groups were varied at ortho, meta and para-position of the phenyl ring to acquire the optimum results.

The results demonstrated the following assumptions about the structural activity relationship, it is evident, that in a group of compounds having -H-, 4-CH_3_, 4-OH, 3-OCH_3_, indole, quinoline, 2-CF_3_, 3-OH, 4-OCH_3_ and 3,4,5 tri-OCH_3_ substituents on phenyl ring (7d-f and 7g-7o) were essentially influencing the anticancer activity significantly, particularly, the compounds substituted with halogens on phenyl ring was (7a and 7b-c) were found to be the most active compounds *in vitro* cytotoxicity. The phenyl ring substituted apart from halogen groups slightly lowers the activity. Lower activity was observed even when the phenyl ring substituted with 3,4,5 tri-OCH_3_ (7i). Whereas electron releasing in groups substituted on phenyl ring were found to be less active against cancers cell lines.

Overall, it can be hypothesized that compounds bearing halogens substituent on phenyl ring have shown potent anticancer activity. In fact, the ortho substituents were found to reduce or diminish the overall activity of the compound. Hence, it can also be hypothesized that steric hindrance might have also played an important role in influencing the activity of the compound. The results from the preliminary structure activity analysis have led to the determination of some key structural requirements for the FQT hybrids to exert their anticancer activity, providing insights into further structural modifications.

## CONCLUSION

The present research reports the successful synthesis, characterization, cytotoxic, DNA cleavage and pharmacokinetic study of new furan, quinoline and 1,2,4-triazole derivatives.

Attempts have been made to predict *in-silico* ADMET/SAR of synthesized molecules. All compounds were found to be in the acceptable range. The compounds 7(a-o) were investigated for DNA cleavage study and analogs of triazole were found to possess significant DNA cleavage activity. Further, triazoles were screened for *in vitro* cytotoxic study on different human cancer cell lines. Four of the tested compounds 7a-c and 7k were effective against A375 and MDA-MB-231 with IC_50_ values of 2.9, 4.0, 7.8 and 5.1 µg/ml; and 6.2, 9.5, 11.3 and 7.3 µg/ml, respectively. The obtained results suggest that these compounds may serve as lead chemical entities for further modification in the search for new classes of potential anticancer agents.

## Figures and Tables

**Fig. (2):**
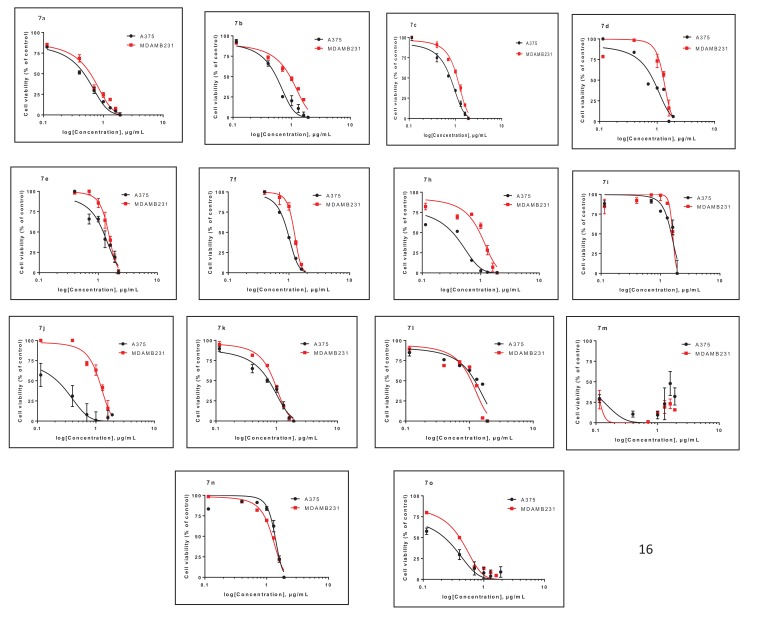
Cell cytotoxic effect of compounds. Log dose response curves were generated in Graph Pad Prism7. The doses of compound (x-axis) were log transformed and then a non-linear curve was fitted to the data using the ‘log (agonist) *versus* normalized response with variable slope’ algorithm. Data represent the mean ± standard deviations of three repeats.

**Table 1:** LD_50_ ADME-TOX Parameters and probability of health effects of substituted 2-(1-furan-2-yl)-4-(5-phenyl-4*H*-1,2,4-triazol-3-yl) quinoline using ACD/ I-Lab 2.0.

**Ligands**	**Intraperitoneal**	**Oral**	**Intravenous**	**Subcutaneous**
**7a**	710(0.27)	790(0.21)	79(0.22)	50(0.45)
**7b**	490(0.4)	970(0.31)	40(0.37)	820(0.36)
**7c**	570(0.4)	820(0.28)	43(0.35)	1100(0.35)
**7d**	760(0.36)	650(0.21)	49(0.32)	1400(0.28)
**7e**	450(0.4)	1000(0.32)	40(0.36)	850(0.38)
**7f**	400(0.41)	880(0.29)	33(0.36)	840(0.36)
**7g**	420(0.39)	1100(0.31)	29(0.36)	790(0.37)
**7h**	430(0.4)	990(0.32)	37(0.36)	860(0.37)
**7i**	470(0.39)	1100(0.28)	41(0.37)	760(0.3)
**7j**	520(0.32)	1000(0.3)	28(0.16)	460(0.29)
**7k**	510(0.2)	300(0.0)	45(0.19)	19(0.36)
**7l**	670(0.34)	720(0.25)	24(0.29)	330(0.35)
**7m**	550(0.38)	1300(0.41)	39(0.35)	980(0.34)
**7n**	540(0.39)	1100(0.37)	35(0.34)	750(0.38)
**Pyrazinamide**	2000(0.83)	540(0.28)	170(0.56)	1000(0.71)
**Ciprofloxacin**	930(0.72)	3500(0.78)	120(0.86)	1400(0.58)
**Streptomycin**	310(0.76)	880(0.53)	110(0.67)	400(0.52)

Estimated LD_50_-mouse value in mg / kg after Intraperitoneal, Oral, Intravenous and Subcutaneous administration. The drugs with moderate effect on reliability index (>0.5). The drugs with border line effect on reliability index (>0.3, <0.5).

**Table 2:** ADME and pharmacological parameters prediction for the ligands substituted 2-(1-furan-2-yl)-4-(5-phenyl-4*H*-1,2,4-triazol-3-yl) quinoline using ADMET/SAR.

**Ligands**	**PlogBB^a^**	**PCaco^b^**	**logHIA^c^**	**logpGI (Non-Substrate)^d^**	**logpGI (Non-Inhibitor)^e^**	**PlogS^f^**	**logpapp^g^**
**7a**	0.9835	0.5000	1.0000	0.8668	0.9113	-3.0191	1.3623
**7b**	0.9835	0.5000	1.0000	0.8668	0.9113	-3.0191	1.3623
**7c**	0.9867	0.5053	1.0000	0.8623	0.9033	-2.9650	1.3260
**7d**	0.9850	0.5440	1.0000	0.8667	0.8749	-3.5746	1.5276
**7e**	0.9751	0.5276	1.0000	0.7918	0.8490	-3.4071	1.4645
**7f**	0.9037	0.5989	1.0000	0.8280	0.6705	-3.5041	1.4564
**7g**	0.9400	0.5597	1.0000	0.7963	0.7894	-3.8666	1.3829
**7h**	0.8150	0.5166	1.0000	0.8118	0.8213	-3.5165	1.3111
**7i**	0.9238	0.5394	1.0000	0.8096	0.8990	-3.6950	1.6543
**7j**	0.9789	0.5096	1.0000	0.8660	0.9316	-2.8715	1.3218
**7k**	0.9672	0.5480	1.0000	0.8628	0.8707	-2.9824	1.6034
**7l**	0.9812	0.5521	1.0000	0.8707	0.8829	-3.7053	1.5793
**7m**	0.9369	0.5219	1.0000	0.8437	0.8589	-3.7860	1.6637
**7n**	0.9808	0.5329	1.0000	0.8529	0.8219	-4.1954	1.4859
**7o**	0.9495	0.5871	1.0000	0.7959	0.7011	-3.4370	1.1852
**Pyrazinamide**	0.9745	0.7222	0.9813	0.8760	0.9731	-0.8476	1.3021
**Ciprofloxacin**	0.9655	0.8956	0.9795	0.9116	0.9231	-3.4638	0.8090
**Streptomycin**	0.9712	0.6968	0.8824	0.8177	0.9230	-2.0122	-0.5128

^a^Predicted blood / brain barrier partion coefficient (1-high penetration, 2- medium penetration and 3- low penetration). ^b^predicted Caco-2 cell permeability in nm / s (acceptable range -1 is poor, +1 is great). ^c^predicted human intestinal absorption in nm / s (acceptable range 0 is poor, >1 is great). ^d^predictedP-glycoprotein Substrate in nm / s (acceptable range of -5 is poor, 1 is great). ^e^predictedP-glycoprotein inhibitor in nm / s (acceptable range 0-1). ^f^predictedaqueous solubility, (Concern value is 0-2 highly soluble). ^g^predictedCaco-2 cell Permeability in cm / s (Concern value is -1 to 1).

**Table 3:** Cell cytotoxic effect of novel 2-(1-furan-2-yl)-4-(5-phenyl-4*H*-1,2,4-triazol-3-yl) quinoline and its analogues 7(a-o).

**Entry**	**A375**		**MDA-MB-231**	
	IC_50_ (µg/mL) ±SD	**R Square**	IC_50_ (µg/mL) ±SD	R square
**7a**	2.9±0.249	0.985	6.2±1.09	0.979
**7b**	4.0±0.39	0.972	9.5±1.17	0.97
**7c**	7.8±0.41	0.981	11.3±1.11	0.985
**7d**	9.9±0.12	0.946	45.6±0.135	0.989
**7e**	31.6±0.64	0.998	74.1±2.21	0.995
**7f**	6.4±0.38	0.977	13.1±1.18	0.950
**7g**	NA	0.921	173.3±2.03	0.967
**7h**	6.1±1.30	0.91	32.3±1.55	0.944
**7i**	29.8±0.40	0.928	88.6±1.10	0.912
**7j**	79.3±0.89	0.975	>320	0.994
**7k**	5.1±1.33	0.907	7.3±1.17	0.921
**7l**	85.1±1.12	0.950	18.2±1.58	0.935
**7m**	207.1±2.03	0.954	270.0±1.20	0.987
**7n**	79.3±1.40	0.948	57.1±2.00	0.994
**7o**	19.6±0.19	0.994	8.6±0.37	0.988
**Cisplatin**	1.3±0.19	0.994	5.1±0.37	0.988

A375, human metastatic melanoma cell line; MDA-MB-231, human breast cancer cell line; NA, no activity; IC_50_, 50% inhibitory concentration; SD, standard deviation of mean, IC_50_ values of compound were evaluated using GraphPad Prism 7 software. Data represent the mean ± standard deviations of three replicates (n=3). **R**-**squared** is a statistical measure of how close the data are to the fitted regression line in graph.
